# Icariin Prevents Amyloid Beta-Induced Apoptosis via the PI3K/Akt Pathway in PC-12 Cells

**DOI:** 10.1155/2015/235265

**Published:** 2015-01-29

**Authors:** Dongdong Zhang, Zhe Wang, Chenxia Sheng, Weijun Peng, Shan Hui, Wei Gong, Shuai Chen

**Affiliations:** ^1^Department of Integrated Chinese and Western Medicine, The Second Xiangya Hospital, Central South University, Changsha, Hunan 410011, China; ^2^Department of Traditional Chinese Medicine, Zhong Shan Hospital Xiamen University, Xiamen, Fujian 361004, China

## Abstract

Icariin is a prenylated flavonol glycoside derived from the Chinese herb *Epimedium sagittatum* that exerts a variety of pharmacological activities and shows promise in the treatment and prevention of Alzheimer's disease. In this study, we investigated the neuroprotective effects of icariin against amyloid beta protein fragment 25–35 (A*β*_25–35_) induced neurotoxicity in cultured rat pheochromocytoma PC12 cells and explored potential underlying mechanisms. Our results showed that icariin dose-dependently increased cell viability and decreased A*β*_25–35_-induced apoptosis, as assessed by MTT assay and Annexin V/propidium iodide staining, respectively. Results of western blot analysis revealed that the selective phosphatidylinositol 3-kinase (PI3K) inhibitor LY294002 suppressed icariin-induced Akt phosphorylation, suggesting that the protective effects of icariin are associated with activation of the PI3K/Akt signaling pathway. LY294002 also blocked the icariin-induced downregulation of proapoptotic factors Bax and caspase-3 and upregulation of antiapoptotic factor Bcl-2 in A*β*_25–35_-treated PC12 cells. These findings provide further evidence for the clinical efficacy of icariin in the treatment of Alzheimer's disease.

## 1. Introduction

Alzheimer's disease (AD), the leading cause of dementia, is characterized by the progressive loss of memory and other cognitive functions [1]. Despite considerable progress towards understanding the complex molecular mechanisms underlying AD, effective treatments able to prevent, halt, or reverse the pathobiology of AD are still lacking [2]. As the global population ages, the economic impact of dementia is expected to exceed that of cancer, heart disease, and stroke combined [3].

According to the amyloid hypothesis, neurodegeneration and dementia in AD are caused by extensive accumulation of amyloid beta peptide (A*β*) in the cerebrum [4, 5]. Extracellular deposits of the A*β* peptide form diffuse and neuritic plaques, and the microtubule assembly protein tau becomes hyperphosphorylated, accumulating intracellularly as neurofibrillary tangles [1]. In addition, widespread loss of neurons and synapses occurs [6]. Numerous studies have demonstrated that apoptosis is the primary mechanism underlying A*β*-induced neuronal death [7–9]; therefore, drugs blocking apoptosis may be useful to prevent neuronal cell death and treat AD [8, 10, 11]. The phosphatidylinositol 3-kinase (PI3K)/Akt pathway, which functions as a critical regulator of cell apoptosis, is thought to play an important role in neurological diseases such as AD [12, 13].

Icariin, which is a prenylated flavonol glycoside derived from the Chinese herb* Epimedium sagittatum*, exerts a variety of pharmacological activities including antioxidant activity [14], immunoregulation [15], antitumor activity [16, 17], and estrogen-like activities [18, 19]. Icariin appears to be able to cross the blood-brain barrier and provide neuroprotective effects by modulating the cholinergic system and inhibiting A*β* neurotoxicity [20]. Studies in mouse models of AD and age-related cognitive decline have demonstrated that icariin improves cognitive ability and inhibits memory impairment [21, 22]. In addition, Zeng et al. demonstrated that icariin reduces neurotoxicity by inhibiting tau protein hyperphosphorylation [23]; however, the mechanism underlying this effect is unknown and the effect of icariin on A*β*-induced apoptosis, a hallmark of AD, remains unclear. A recent study suggested that icariin inhibits apoptosis via the PI3K/Akt pathway [24]. Therefore, in this study we evaluated the role of PI3K/Akt signaling in the protective effects of icariin against A*β*-induced apoptosis in cultured rat pheochromocytoma PC12 cells. Our results provide further evidence for the clinical efficacy of icariin in the treatment of AD.

## 2. Materials and Methods

### 2.1. Reagents

Icariin (purity > 98%) was purchased from the Hunan Institute for the Control of Pharmaceutical and Biological Products (Changsha, China). The selective PI3K inhibitor LY294002 was purchased from Sigma-Aldrich (St. Louis, MO, USA), and the Annexin-V-FLUOS staining kit was purchased from Roche (Penzberg, Germany). Dulbecco's modified Eagle medium (DMEM), penicillin, and streptomycin were purchased from Solarbio (Shanghai, China), and fetal bovine serum was purchased from Invitrogen/Gibco (Carlsbad, CA, USA). Antibodies against Akt, p-Akt (Ser473), Bax, Bcl-2, caspase-3, and *β*-actin were obtained from Abzoom (Dallas, TX, USA). Anti-mouse horseradish peroxide- (HRP-) conjugated IgG and anti-rabbit HRP-conjugated IgG secondary antibodies were obtained from Santa Cruz Biotechnology (Santa Cruz, CA, USA).

### 2.2. Peptide Preparation

A*β*_25–35_ was dissolved in deionized distilled water at a concentration of 1 mM and incubated at 37°C for 72 h to induce aggregation [23].

### 2.3. Cell Culture and Drug Treatments

PC12 cells were maintained in DMEM containing 10% fetal bovine serum, 100 U/mL penicillin, and 100 U/mL streptomycin at 37°C in a 5% CO_2_ incubator; the medium was changed every other day. The cells were cultured in serum-free medium for 12 h prior to treatment with icariin, and A*β*_25–35_ was added 1 h later. In experiments involving the inhibition of PI3K/Akt signaling, LY294002 (50 *μ*M) was added to the medium 1 h prior to icariin treatment.

### 2.4. Cell Viability Assay

PC12 cells were seeded into 96-well plates (0.5 × 10^4^ cells/well) and assessed for response to A*β*_25–35_ and icariin using the MTT assay. Briefly, the cells were pretreated with icariin (2.5, 5.0, 10.0, or 20.0 *μ*M) or vehicle only for 1 h and then exposed to 20 *μ*M preaggregated A*β*_25–35_ for 24 h in the continued presence of icariin or vehicle. To determine viability, the cells were then treated with 5 mg/mL MTT for 4 h at 37°C and then the medium was carefully removed. The resulting formazan crystals were dissolved in 150 *μ*L dimethyl sulfoxide (DMSO), and absorbance at 570 nm was determined using a plate reader.

### 2.5. Apoptosis Assay

Apoptosis was detected using the Annexin-V-FLUOS staining kit (Roche). Briefly, 5 × 10^5^ cells were collected, resuspended in phosphate buffered saline, and incubated with Annexin V/PI labeling solution for 20 min at room temperature in the dark. The cells were then analyzed by flow cytometry (Becton Dickinson, USA). A minimum of 10,000 events were counted per sample.

### 2.6. Western Blot Analysis

Cells were collected after drug treatment, and whole cell lysates were prepared by incubation in RIPA buffer (Cell Signaling Technology, Boston, MA, USA) supplemented with a protease inhibitor cocktail (Roche), according to the manufacturer's instructions. The lysates (20 *μ*g protein) were separated by SDS-PAGE (6%–12%) and transferred to polyvinylidene fluoride membranes. After blocking the membranes with 5% nonfat dry milk, primary antibodies (1 : 500 dilution) were added, and the membranes were incubated for 2 h at room temperature, followed by incubation with the corresponding anti-rabbit IgG, HRP-linked secondary antibodies at room temperature for 1 h. Extensive washes were performed between each step. Bound antibodies were visualized using the ECL Advance western blotting detection kit (GE Healthcare, Little Chalfont, Buckinghamshire, UK), and images were obtained using the LAS-4000 imaging system (Fuji Film, Tokyo, Japan).

### 2.7. Statistical Analysis

The data are expressed as mean ± standard deviation (S.D.). Groups were compared by Student's *t*-test and analysis of variance; *P* < 0.05 was considered significant.

## 3. Results

### 3.1. Icariin Increased Viability of PC12 Cells Treated with A*β*_25–35_

**Figure 1:**
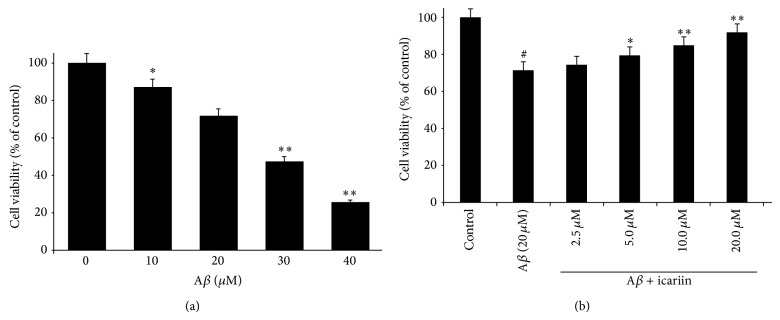
Icariin protects against A*β*_25–35_-induced cytotoxicity in PC12 cells. (a) Effects of A*β*_25–35_ on cell viability. PC12 cells were treated with 10–40 *μ*M A*β*_25–35_, and cell viability was assessed by the MTT assay. (b) Protective effect of icariin against A*β*_25–35_-induced cytotoxicity in PC12 cells. After 1 h pretreatment with icariin (2.5, 5, 10, or 20 *μ*M), PC12 cells were treated with 20 *μ*M A*β*_25–35_ for 24 h. Results are presented as mean ± S.D. of five independent experiments. ^*^ *P* < 0.05; ^**^ *P* < 0.01 versus A*β*_25–35_ treatment; ^#^ *P* < 0.01 versus untreated control.

Because A*β* has been shown to induce neuronal apoptosis in the pathogenesis of AD, we evaluated the effect of A*β*_25–35_ on PC12 cell viability. Cultures were treated with 10, 20, 30, or 40 *μ*m A*β*_25–35_, and cell viability was determined 24 h later using the MTT assay. As shown in Figure 1, 20 *μ*m A*β*_25–35_ significantly decreased PC12 cell viability, and treatment with 40 *μ*m A*β*_25–35_ resulted in the minimal survival of 25.5% compared with vehicle-treated controls, indicating a dose-dependent effect.

To evaluate the effects of icariin on A*β*_25–35_-induced cytotoxicity, we pretreated PC12 cells with 2.5, 5.0, 10.0, or 20.0 *μ*m icariin 1 h prior to 24 h treatment with 20 *μ*m A*β*_25–35_. Our results show that, at doses ≥5.0 *μ*m, icariin protected cells against the toxic effects of A*β*_25–35_ (Figure 1). Thus, icariin attenuates the effects of A*β*_25–35_ on cell viability in a dose-dependent manner.

### 3.2. Icariin Decreased A*β*_25–35_-Induced Apoptosis in PC12 Cells

**Figure 2:**
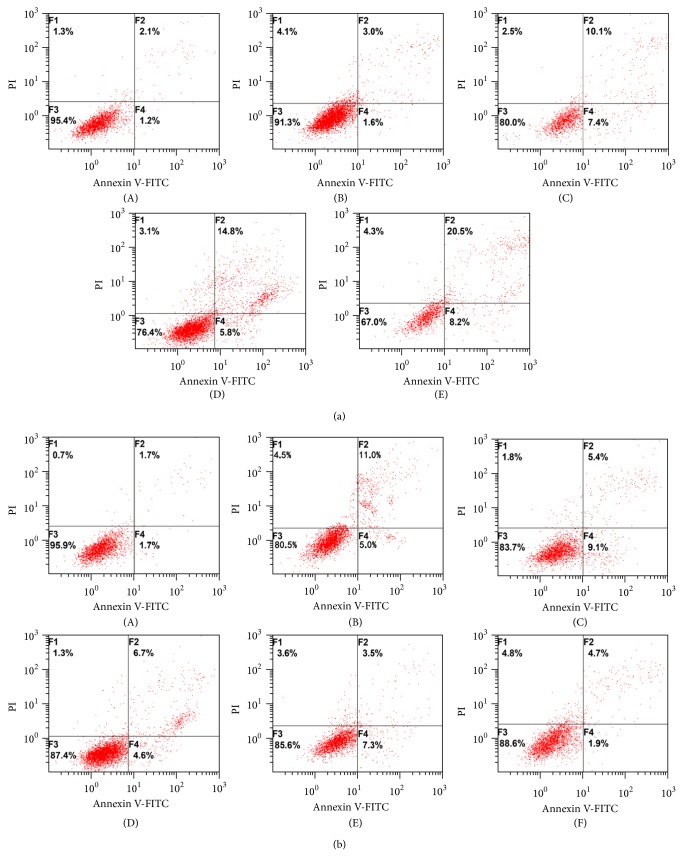
Protective effects of icariin on A*β*_25–35_-induced apoptosis in PC12 cells. (a) Proapoptotic effect of A*β*_25–35_. After treatment with the following concentrations of A*β*_25–35_, cell apoptosis was evaluated by Annexin V/PI staining: (A) 0 *μ*M, (B) 10 *μ*M, (C) 20 *μ*M, (D) 30 *μ*M, or (E) 40 *μ*M. (b) Protective effect of icariin against A*β*_25–35_-induced apoptosis. After 1 h pretreatment with icariin (0, 2.5, 5, 10, or 20 *μ*M), PC12 cells were treated with 20 *μ*M A*β*_25–35_ for 24 h. Apoptosis was evaluated by Annexin V/PI staining. (A) Untreated control, (B) 20 *μ*M A*β*_25–35_ only, (C) 2.5 *μ*M icariin, (D) 5 *μ*M icariin, (E) 10 *μ*M icariin, and (F) 20 *μ*M icariin. Apoptosis rates are shown in Supplementary Figure  1 (available online at http://dx.doi.org/10.1155/2015/235265).

The proapoptotic effects of A*β*_25–35_ were then assessed by Annexin V/PI double staining. Our data showed that 10 *μ*m A*β*_25–35_ was sufficient to induce PC12 cell apoptosis (Figure 2). Consistent with results of the MTT assay, A*β*_25–35_ increased apoptosis in a dose-dependent manner.

Results of Annexin V/PI staining revealed that icariin is able to prevent A*β*_25–35_-induced apoptosis in PC12 cells. As shown in Figure 2, 20 *μ*m A*β*_25–35_ increased both early and late apoptosis in PC12 cells, with a total apoptosis rate of 15.8% by 24 h. However, pretreatment with icariin for 1 h prior to A*β*_25–35_ exposure decreased the apoptosis rate to 15.0% (2.5 *μ*m icariin), 11.0% (5.0 *μ*m), 10.2% (10 *μ*m), and 6.6% (20 *μ*m), respectively. The apoptosis rate of cells treated with 2.5 *μ*M icariin did not differ significantly from that of controls treated with A*β*_25–35_ only. These results indicate that icariin dose-dependently suppresses A*β*_25–35_-induced apoptosis.

### 3.3. Role of PI3K/Akt Signaling in the Protective Effect of Icariin against A*β*_25–35_-Induced Apoptosis

**Figure 3:**
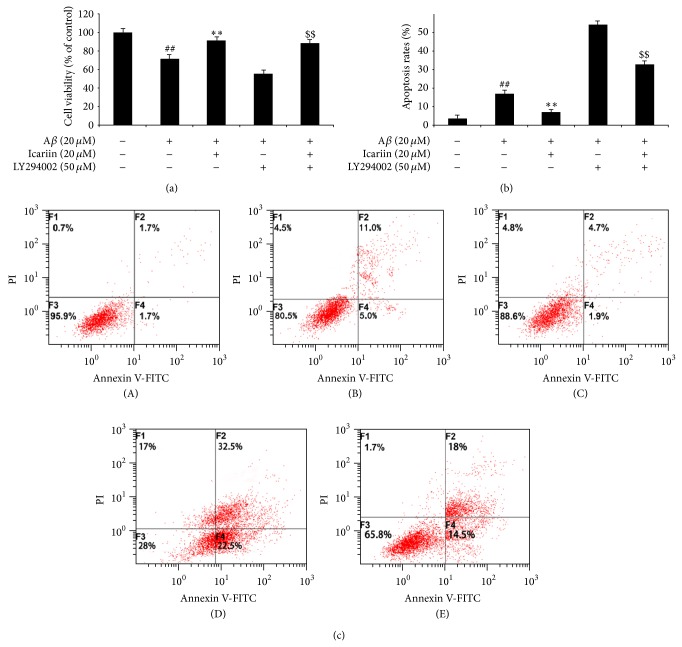
Involvement of PI3K/Akt signaling in the protective effects of icariin against A*β*_25–35_-induced cytotoxicity in PC12 cells. After 1 h pretreatment with the PI3K inhibitor LY294002 (50 *μ*M), PC12 cells were treated with 20 *μ*M icariin for 1 h, followed by A*β*_25–35_ treatment for 24 h. Cell viability and apoptosis were determined by MTT assay and Annexin V/PI double staining, respectively. (a) LY294002 inhibited the icariin-mediated increase in viability in A*β*_25–35_-treated cells. (b) LY294002 inhibited the icariin-mediated decrease in apoptosis in A*β*_25–35_-treated cells. (c) (A) Untreated control, (B) A*β*_25–35_ treatment, (C) icariin treatment, (D) LY294002 treatment, and (E) icariin + LY294002. Results are presented as mean ± S.D. of triplicate independent experiments. ^##^ *P* < 0.01 versus untreated control; ^**^ *P* < 0.01 versus A*β*_25–35_ treatment; ^$$^ *P* < 0.01 versus icariin treatment.

PI3K/Akt signaling plays a pivotal role in cell survival, and its activation may have antiapoptotic effects. We therefore treated PC12 cells with PI3K inhibitor LY294002 (50 *μ*m) and found the LY294002 increased apoptosis and decreased viability in PC12 cells treated with A*β*_25–35_ (Figure 3). This result suggests that the PI3K/Akt signaling pathway is involved in A*β*_25–35_-induced apoptosis. To confirm the role of PI3K/Akt signaling in the neuroprotective effects of icariin, PC12 cells were pretreated with 50 *μ*mL Y294002 for 30 min, followed by 30 min treatment with icariin (20 *μ*m) and then 24 h treatment with 20 *μ*m A*β*_25–35_. Our results show that LY294002 diminished the effects of icariin on A*β*_25–35_-induced apoptosis.

### 3.4. Icariin Activates the PI3K/Akt Signaling Pathway through Akt Activation

**Figure 4:**
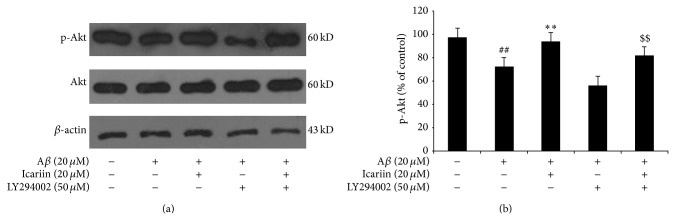
Icariin inhibits A*β*_25–35_-induced cytotoxicity through the activation of PI3K/Akt signaling. After 1 h pretreatment with the PI3K inhibitor LY294002 (50 *μ*M), PC12 cells were treated with 20 *μ*M icariin for 1 h, followed by A*β*_25–35_ treatment for 24 h. Phosphorylation of Akt was assessed by western blot. Densitometry values represent the mean ± S.D. of triplicate independent experiments. ^##^ *P* < 0.01 versus untreated control; ^**^ *P* < 0.01 versus A*β*_25–35_ treatment; ^$$^ *P* < 0.01 versus icariin treatment.

To further investigate the role of PI3K/Akt signaling in the protective effects of icariin against A*β*_25–35_-induced apoptosis, we evaluated Akt activation by western blot analysis using phosphorylation state-specific antibodies. A previous study reported that icariin suppresses apoptosis by activating the PI3K/Akt pathway through the phosphorylation of Akt (Ser473) [23]. Our results show that A*β*_25–35_ significantly inhibited Akt phosphorylation, but pretreatment with icariin attenuated the effects of A*β*_25–35_, indicating that icariin activates the PI3K/Akt pathway (Figure 4). LY294002 significantly inhibited the effects of icariin treatment, providing additional evidence for the role of PI3K/Akt signaling in the protective effects of icariin.

### 3.5. Icariin Attenuates Bax and Caspase-3 Expression through PI3K/Akt Signaling

**Figure 5:**
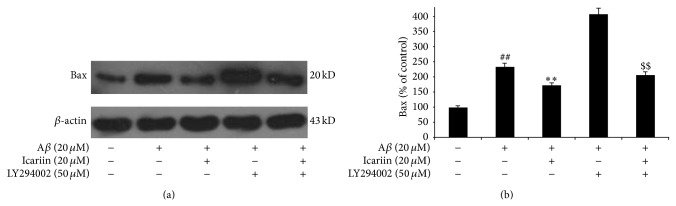
Icariin decreased Bax expression in PC12 cells after exposure to A*β*_25–35_. After pretreatment with 50 *μ*M LY294002 for 1 h, PC12 cells were treated with 20 *μ*M icariin for 1 h, followed by A*β*_25–35_ treatment for 24 h. Densitometry values are presented as the mean ± S.D. of triplicate independent experiments. ^##^ *P* < 0.01 versus untreated control; ^**^ *P* < 0.01 versus A*β*_25–35_ treatment; ^$$^ *P* < 0.01 versus icariin treatment.

**Figure 6:**
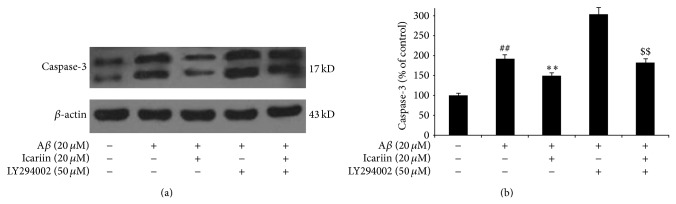
Icariin decreased caspase-3 expression in PC12 cells after exposure to A*β*_25–35_. After pretreatment with 50 *μ*M LY294002 for 1 h, PC12 cells were treated with 20 *μ*M icariin for 1 h, followed by A*β*_25–35_ treatment for 24 h. Densitometry values are presented as the mean ± S.D. of triplicate independent experiments. ^##^ *P* < 0.01 versus control; ^**^ *P* < 0.01 versus A*β*_25–35_ treatment; ^$$^ *P* < 0.01 versus icariin treatment.

The major executioners of apoptosis are the proteases caspase-3 and Bax. Activation of PI3K/Akt directly and indirectly induces the phosphorylation of Bax and caspase-3, promoting their interactions with other proteins and inhibiting apoptotic activity. As shown in Figures 5 and 6, protein levels of caspase-3 and Bax were significantly increased by A*β*_25–35_ treatment, compared with untreated controls. However, results of western blotting show that icariin decreased levels of caspase-3 and Bax in A*β*_25–35_-treated cells. PI3K inhibitor LY294002 attenuated the effects of icariin, indicating that icariin inhibits expression of Bax and caspase-3 through the activation of PI3K/Akt signaling.

### 3.6. Icariin Stimulates Bcl-2 Expression via the PI3K/Akt Pathway

**Figure 7:**
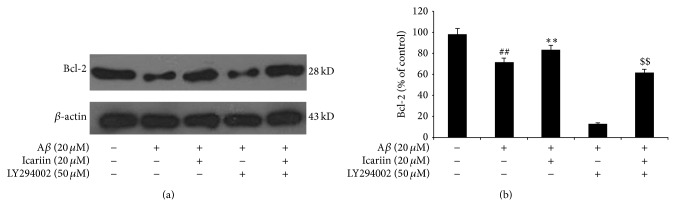
Icariin increased Bcl-2 protein levels in PC12 cells exposed to A*β*_25–35_. After pretreatment with 50 *μ*M LY294002 for 1 h, PC12 cells were treated with 20 *μ*M icariin for 1 h, followed by 24 h A*β*_25–35_ treatment. Densitometry values are presented as mean ± S.D. of triplicate independent experiments. ^##^ *P* < 0.01 versus control; ^**^ *P* < 0.01 versus A*β*_25–35_ treatment; ^$$^ *P* < 0.01 versus icariin treatment.

Because activation of the PI3K/Akt pathway is known to upregulate Bcl-2 and prevent apoptosis, we next evaluated the effects of icariin on Bcl-2 expression. As shown in Figure 7, icariin attenuated the A*β*_25–35_-induced decrease in Bcl-2 protein levels, but the effect of icariin on Bcl-2 was diminished by LY294002. This result provides further evidence for the role of PI3K/Akt signaling in the antiapoptotic effects of icariin.

## 4. Discussion

Extensive cell death is one of the most important pathological manifestations of AD [25–28]. Although the causal events remain unclear, preventing neuronal cell death is a promising approach to treating AD [29]. The purpose of this study was to better understand the neuroprotective effects of icariin against A*β* neurotoxicity and identify the signaling pathways involved. Because activation of the PI3K/Akt pathway promotes cell survival [12], we assessed the role of PI3K/Akt signaling in the protective effects of icariin. First, we demonstrated that A*β*_25–35_ decreased PC12 cell viability and increased apoptosis in a dose-dependent manner. Pretreatment with icariin dose-dependently increased viability and decreased apoptosis in A*β*_25–35_-treated cells. These findings are consistent with a previous study reporting that icariin prevents cell death associated with A*β* toxicity [23].

The PI3K/Akt signaling pathway plays a crucial role in cell survival by inhibiting apoptosis [30, 31]. Recent studies suggest that activation of PI3K/Akt signaling attenuates A*β*-induced apoptosis through the inhibition of glycogen synthase kinase-3 beta, which suppresses tau protein hyperphosphorylation and the formation of neurofibrillary tangles [32, 33]. Recent studies have also reported that icariin inhibits corticosterone-induced apoptosis [24] and lipopolysaccharide-induced inflammation through the PI3K/Akt pathway [34]. Cai et al. suggested that the protective effects of icariin against A*β* neurotoxicity depend on the insulin/insulin-like growth factor 1 pathway [35], which is upstream of the PI3K/Akt pathway [36]. Our results demonstrating that icariin increased viability and decreased apoptosis in A*β*-treated PC12 cells and that these effects were attenuated by a PI3K inhibitor strongly support a role for this signaling pathway in icariin-mediated neuroprotection.

Because icariin has been reported to activate PI3K/Akt signaling through the phosphorylation of Akt (Ser473) [23], we evaluated Akt phosphorylation in icariin-treated PC12 cells. The results of western blot analysis confirmed that icariin significantly increased phosphorylation of Akt at Ser473, and that this effect was suppressed by LY294002, providing further evidence that icariin suppresses apoptosis through activation of PI3K/Akt signaling.

PI3K/Akt signaling promotes cell survival by direct or indirect interaction with proteins associated with apoptosis [12], such as Bcl-2, which promotes cell survival [37], and Bax, which promotes mitochondrial permeability leading to apoptosis [13]. Activated Akt directly or indirectly suppresses the apoptotic activity of Bax by serine phosphorylation [13]. Caspase-3 is activated by exposure to A*β* peptides, contributing to the pathophysiology of AD through cell death-independent mechanisms, as well as apoptosis [38, 39]. We found that icariin attenuated the effects of A*β*_25–35_ in PC12 cells, increasing protein levels of prosurvival factor Bcl-2 and decreasing protein levels of proapoptotic factors Bax and caspase-3. PI3K inhibitor LY294002 suppressed these effects of icariin.

The extracellular signal-regulated protein kinase (ERK) pathway may also play an important role in the antiapoptotic effects of icariin. ERK, which is a converging point of multiple signal transduction pathways involved in apoptosis [40], has been shown to decrease* bcl*-2 gene expression through the tumor suppressor p53 [40]. In addition, Akt may act synergistically with p90 ribosomal S6 kinase to inhibit the proapoptotic activity of Bax through phosphorylation [13]. Although several studies have reported that icariin promotes cell proliferation by activating ERK [41, 42], another study suggested that icariin inhibits corticosterone-induced apoptosis in primary cultured rat hippocampal neurons by blocking p38 mitogen-activated protein kinase but not ERK [43]. Therefore, further research is needed to clarify the role of ERK in the neuroprotective effects of icariin.

In conclusion, this study provides evidence that icariin is effective in suppressing A*β*_25–35_-induced apoptosis in PC12 cells, which likely occurs through the activation of PI3K/Akt signaling. Our results also indicate that icariin upregulates Bcl-2 and downregulates Bax, two critical downstream effectors in PI3K/Akt signaling. Further studies are needed to better understand the cellular mechanisms underlying the neuroprotective effects of icariin, which may ultimately lead to the development of a novel therapy for the treatment of AD.

## Supplementary Material

Supplementary figure 1: Protective effects of icariin on Aß25–35 induced apoptosis in PC12 cells.(I) Pro-apoptotic effect of Aß25–35. After treatment with the following concentrations of Aß25–35,cell apoptosis was evaluated by AnnexinV/PI staining. (II) Protective effect of icariin against Aß25-35-induced apoptosis. After 1h pretreatment with icariin (0, 2.5, 5, 10,or 20 µM), PC12 cells were treated with 20 µM Aß25–35 for 24h. Apoptosis was evaluated by AnnexinV/PI staining. Results are presented as the mean ±S.D. of five independent experiments.∗∗p <0.01 versus Aß25–35 treatment; # p <0.05 versus untreated control.
