# Supplementary material for: Icariin Prevents Amyloid Beta-Induced Apoptosis via the PI3K/Akt Pathway in PC-12 Cells
Source: Evid Based Complement Alternat Med. 2015 Jan 29;2015:235265. doi: 10.1155/2015/235265 (PMC4326344; doi:10.1155/2015/235265)
Supplement: Supplementary file 1 — Supplementary figure 1: Protective effects of icariin on Aß25–35 induced apoptosis in PC12 cells.(I) Pro-apoptotic effect of Aß25–35. After treatment with the following concentrations of Aß25–35,cell apoptosis was evaluated by AnnexinV/PI staining. (II) Protective effect of icariin against Aß25-35-induced apoptosis. After 1h pretreatment with icariin (0, 2.5, 5, 10,or 20 µM), PC12 cells were treated with 20 µM Aß25–35 for 24h. Apoptosis was evaluated by AnnexinV/PI staining. Results are presented as the mean ±S.D. of five independent experiments.∗∗p <0.01 versus Aß25–35 treatment; # p <0.05 versus untreated control. [file 235265.f1.pdf]

I

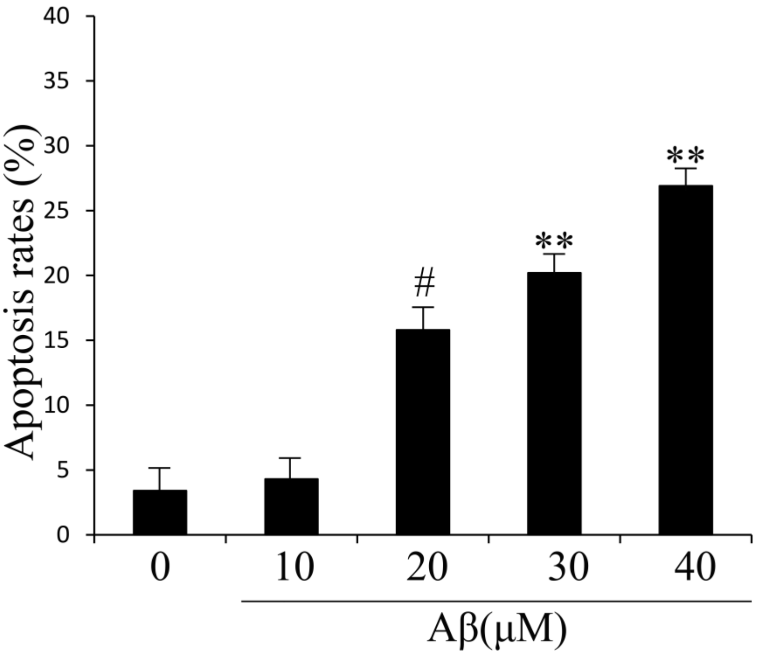

II

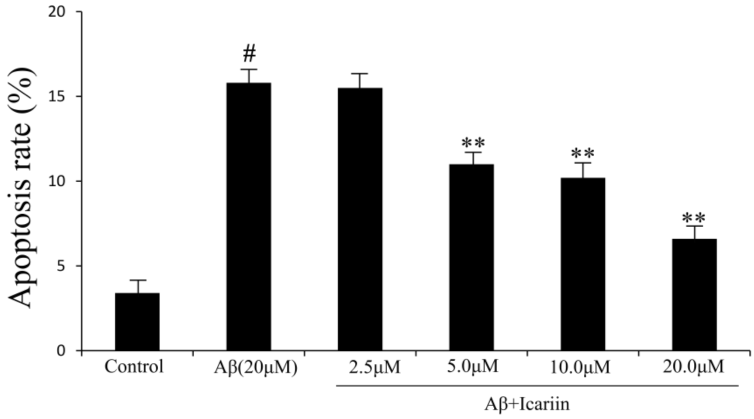

Supplementary Fig1: Protective effects of icariin againstAβ_{25-35} induced apoptosis in PC12 cells.(I) Effects of Aβ_{25-35} on cell apoptosis. PC12 cells were treated with increasing concentrations of Aβ_{25-35},andapoptosis wasevaluatedbyAnnexinV/PI staining.(II)Protective effect of icariin againstA$\beta_{25\text{-}35}$-induced apoptosis. PC12 cells were pretreated with icariin (2.5, 5, 10,or20μM) for 1h, and then treated with 20 μM Aβ_{25-35} for24h. Apoptosis wasevaluatedby AnnexinV/PI staining. Results are presented as the mean ± S.D. of five independent experiments.**p <0.01 versus Aβ_{25-35} treatment; # p <0.05 versus untreated control.
